# Improvement of a low pH antigen-antibody dissociation procedure for ELISA measurement of circulating anti-Aβ antibodies

**DOI:** 10.1186/1471-2202-8-22

**Published:** 2007-03-20

**Authors:** Qingyou Li, Marcia Gordon, Chuanhai Cao, Kenneth E Ugen, Dave Morgan

**Affiliations:** 1 Alzheimer's Research Laboratory, Department of Molecular Pharmacology and Physiology, University of South Florida, Tampa, FL 33612-4799, USA; 2 Department of Molecular Medicine, University of South Florida, Tampa, FL 33612-4799, USA; 3 Byrd Institute for Alzheimer's Disease, Tampa, FL 33612-4799, USA

## Abstract

**Background:**

Prior work from our group found that acid dissociation (pH 2.5 incubation) of serum from APP transgenic mice vaccinated against Aβ increased the apparent anti-Aβ titers, suggesting antibody masking by antigen in the ELISA assay. Subsequently, we found that pH 2.5 incubation of serum from unvaccinated non-transgenic mice showed antibody binding to Aβ1–42, but no increase when other proteins, including shorter Aβ peptides, coated the ELISA plate. To investigate further the effects of low pH incubation on apparent anti-Aβ1–42 signals, we examined normal sera from nonTg unvaccinated mice, nonTg mice vaccinated with Aβ peptide (to produce authentic anti-Aβ antibodies) or a monoclonal antibody against Aβ (6E10) using competitive-inhibition ELISA and Aβ epitope mapping assays. In addition, we examined use of a less stringent low pH procedure at pH 3.5, to ascertain if it had the same effects as the pH 2.5 procedure.

**Results:**

We believe there are three distinct effects of pH 2.5 incubation.; A) an artifactual increase in binding to full length Aβ by mouse immunoglobulin which has low affinity for Aβ, B) an inactivation of anti-Aβ antibodies that is time dependent and C) unmasking of high affinity anti-Aβ antibodies when high levels of circulating Aβ is present in APP transgenic mice. All three reactions can interact to produce the final ELISA signal. Incubation of sera from unvaccinated nonTg mice at pH 2.5 enhanced ELISA signals by process A. Conversely, pH 2.5 incubation of sera from vaccinated nonTg mice with caused a time dependent reduction of antibody signal by process B (overcoming the increase caused by A). The artifactual anti-Aβ ELISA signal enhanced by pH 2.5 incubation of normal mouse sera could not be effectively competed by low to moderate concentrations of Aβ, nor bind to shorter Aβ peptides in a manner similar to authentic anti-Aβ antibodies. Incubation of mouse sera at pH 3.5 caused neither an apparent increase in anti-Aβ ELISA signal, nor an inactivation of the ELISA signals resulting from either vaccination or monoclonal antibodies. However, incubation at pH 3.5 was able to completely reverse the reduction in ELISA signal caused by Aβ complexing with antibodies in sera from vaccinated mice or monoclonal anti-Aβ antibodies.

**Conclusion:**

Incubation at pH 3.5 is sufficient to dissociate Aβ bound to anti-Aβ antibodies without producing artifactual increases in the signal, or inactivating authentic antibody binding. Thus, use of pH 3.5 is a considerable improvement over pH 2.5 incubation for unmasking anti-Aβ antibodies in ELISA assays to measure antibodies in APP transgenic mouse sera.

## Background

It has been well documented that active immunization of transgenic mice against the Aβ peptide can dramatically reduce the deposition of amyloid [1] and improve learning and memory behavior in amyloid depositing transgenic mice[2,3]. However, the anti-Aβ antibody response to vaccination of amyloid precursor protein (APP) transgenic mice appeared lower than in nontransgenic (nonTg) littermates [4-6]. It has also been reported in some studies that patients with Alzheimer disease (AD) have lower levels of serum anti-Aβ antibodies than healthy age-matched individuals [7,8], although others report no change [9] or an increase [10].

This impaired antibody response may result from self tolerance in the APP transgenic mice, or absorption of the serum antibody by circulating Aβ, which interferes with antibody binding to Aβ coated ELISA plates (these are not mutually exclusive). This latter explanation was supported by our prior work, where acid dissociation of serum increased the apparent anti-Aβ antibody titer in vaccinated APP transgenic mice [5]. Subsequently, we unexpectedly found that the pH 2.5 dissociation procedure also enhanced ELISA signals against Aβ1–42 in normal sera from nonTg unvaccinated mice. This suggested that either a) normal mice have measurable anti-Aβ antibody titers that are masked by the low levels of circulating murine Aβ or b) pH 2.5 incubation produces nonspecific interactions between IgG and human Aβ1–42.

We have performed over 100 experiments in the last 3 years in order to identify which of these options is correct. Our data now convince us A) pH 2.5 incubation does produce an artifactual association between IgG and full length Aβ that is not due to typical antigen-antibody interactions. In addition, we find that B) pH 2.5 incubation also causes inactivation of authentic anti-Aβ antibodies formed by vaccination. Finally, C) the low pH incubation can unmask antibody bound to Aβ in circulation. Our prior data are now explained in terms of all three events occurring simultaneously. In addition, we demonstrate that use of pH 3.5 instead of pH 2.5 can eliminate the artifactual binding and the antibody inactivation, while retaining the antibody-antigen dissociation required to assess the true antibody titers in serum.

## Results

### An increased anti-Aβ ELISA signal in sera from vaccinated transgenic mice with preincubation at pH 2.5 is also found in sera from nonTg unvaccinated mice

**Figure 1:**
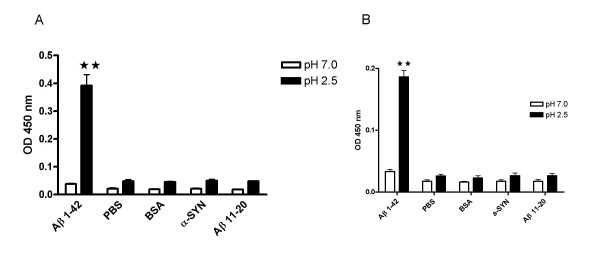
Incubation at pH 2.5 induced increase in anti-Aβ ELISA signal is present not only in sera from vaccinated mice, but also untreated mice. Sera were collected from APP transgenic mice vaccinated (Vax +) with Aβ1–42 (panel A) and nonTg unvaccinated (Vax -) mice (panel B; normal serum), preincubated with dissociation buffer at either pH 7 (open bars) or pH 2.5 (solid bars) at room temperature for 20 minutes and centrifuged through 10,000 MW filters, brought to neutral pH and reconstituted to the original serum dilution volume. The sera were then incubated on ELISA plates coated with one of the following at 5 μg/ml; human Aβ1–42, phosphate buffered saline (PBS; a "no protein" control), bovine serum albumin (BSA), recombinant alpha-synuclein (a-SYN) or Aβ peptide amino acids 11–20. ELISA assays were completed as described in methods and the optical density (OD) at 450 nm used to estimate the amount of antibody binding to the ELISA plate. Note different Y-axis scales in panels A and B. Results are presented as mean ± SEM. ** P < 0.001 compared to pH 7.0.

As observed previously, preincubation at pH 2.5 increases the ELISA signal against Aβ1–42 when sera are taken from APP transgenic mice that have been vaccinated against the Aβ peptide (fig 1A). This enhanced signal was not observed when other proteins or PBS were used to coat the ELISA plate. We had interpreted this as indicating that the low pH incubation dissociated antigen antibody complexes formed in the serum of vaccinated mice overproducing Aβ (due to APP transgenesis). However, when we performed the same pH 2.5 preincubation using normal sera from nonTg mice (thus not overproducing the Aβ peptide) who had never been vaccinated against Aβ (thus, presumed to have very low anti-Aβ titers), we observed a similar, albeit smaller, increase in the apparent anti-Aβ titer (fig. 1B; note difference in Y axis values). Moreover, this also was specific for Aβ1–42 relative to other proteins. This latter signal we now believe is due to the artifactual binding of mouse immunoglobulin to Aβ1–42 after incubation at pH 2.5 (process A).

### pH 2.5 induced artifactual anti-Aβ ELISA signal appears due to IgG rather than other serum proteins

**Figure 2:**
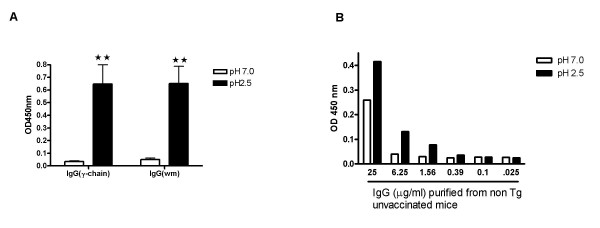
Incubation at pH 2.5 induced artifactual ELISA signal appears to be IgG associated. Panel 2A. Normal sera were collected from nonTg unvaccinated mice, diluted 1:400 and preincubated at either pH 7 (open bars) or pH 2.5 (solid bars) as described in methods. After centrifugation, neutralization and reconstitution of sera volume, the sera were incubated on the ELISA plates coated with human Aβ1–42 for 60 minutes. After washing the plate, anti-mouse gamma chain specific (left) or anti-mouse whole IgG (right) were pipetted into the different wells of plate. Panel 2B. Normal sera from nonTg unvaccinated mice were incubated at pH 7.0 (open bars) or pH 2.5 (solid bars) and then IgG was purified with protein A/G (Pierce) as described in methods. Protein was measured with BCA protein assay Kit (Pierce), and dilutions of the purified IgG were incubated as the indicated concentrations on ELISA plates coated with Aβ1–42. ELISA assays were completed as described in methods. ** P < 0.001 compared to pH 7.0.

In our prior dissociation study, the detection (secondary) antibody was directed against whole-mouse IgG (Sigma, product # A 4416). There is some potential for this detection antibody to react with other Ig classes such as IgM or IgA. To address whether the enhanced ELISA signal from nonTg unvaccinated mice after acid dissociation is specific for IgG, we tested a more specific anti-mouse IgG γ-chain detection antibody (Sigma, product# A 3673). The data showed the ELISA signal detected by anti-mouse IgG γ chain antibody was the same as detected by anti-whole IgG antisera (fig. 2A). In addition, IgG purified from nonTg unvaccinated mice using ImmunoPure (protein A/G) IgG purification Kit retained the enhanced signal towards Aβ on ELISA plates when incubated at pH 2.5. (fig. 2B). These data indicate that artifactual pH 2.5 induced anti-Aβ ELISA signal appears associated with IgG.

### Duration of low pH exposure differentially affects anti-Aβ ELISA signals in sera from unvaccinated mice compared to vaccinated mice

**Figure 3:**
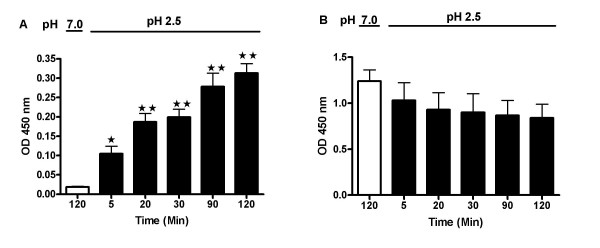
Duration of pH 2.5 exposure differentially affects ELISA signals in sera from unvaccinated nonTg mice compared to vaccinated nonTg mice. Sera were collected from unvaccinated mice (3A) and mice vaccinated with human Aβ1–42 peptide (3B), diluted 1:400. Aliquots were incubated with dissociation buffer at pH 7 (open bar, 120 min) or pH 2.5 (solid bars) for 5, 20, 30, 90 and 120 minutes. The ELISA assays were completed as described in methods and the optical density at 450 nm used to estimate the amount of antibody binding to the ELISA plate. Results are presented as mean ± SEM. * P < 0.05; ** P < 0.001 compared to pH 7.0.

The sera from unvaccinated nonTg mice and nonTg mice vaccinated against Aβ were exposed to dissociation buffer pH at 2.5 for 5, 20, 30, 90 and 120 minutes. The data showed that the ELISA signal of unvaccinated mice (fig. 3A) increased in a time dependent manner when the sera were preincubated at pH 2.5. However, in vaccinated mice which have authentic anti-Aβ antibodies, the antiserum titers raised against human Aβ1–42 peptide were decreased in a time dependent fashion (fig. 3B; also note the different Y axes between the two figures) Thus in the absence of authentic anti-Aβ antibody (3A), low pH artifact (process A) is the only process active and increases the apparent anti-Aβ titer, while in the presence of authentic antibody (3B) the pH 2.5 exposure inactivates antibody (process B) and this overcomes any artifactual increase present in the sera.

### Low pH induced artifactual ELISA signals only bind to full length Aβ; Authentic anti-Aβ antibodies detect both full length Aβ and N terminal fragments

**Figure 4:**
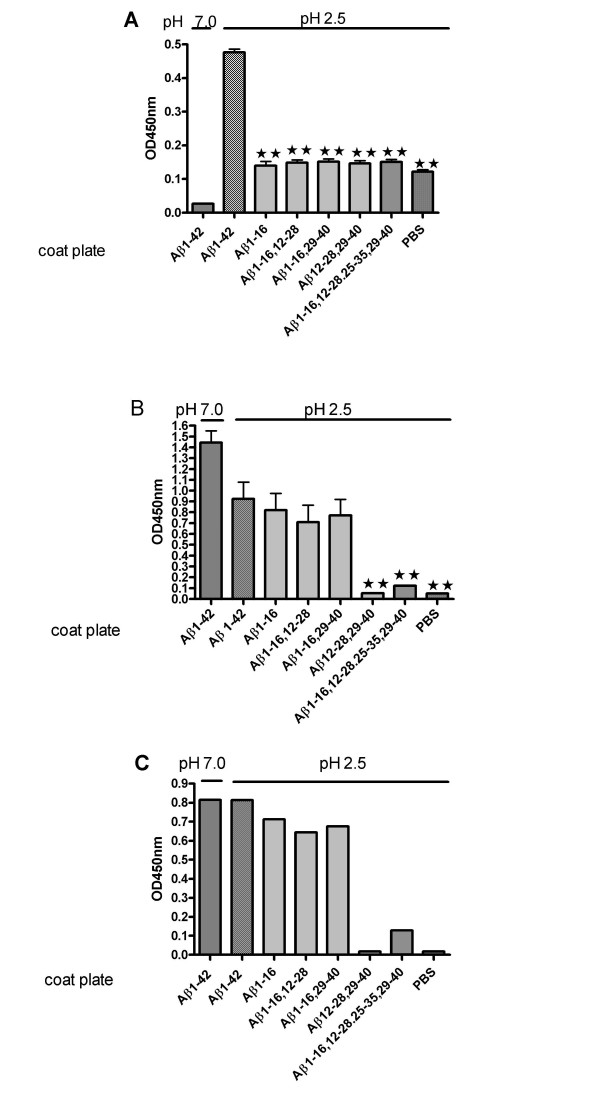
Incubation at pH 2.5 induced artifactual ELISA signal only interacts with full length Aβ; Authentic antibodies bind both full length Aβ and N terminal fragments. Epitope mapping of normal sera from unvaccinated mice (panel A;1:400 dilution), mice vaccinated with Aβ1–42 peptide (panel B; 1:400 dilution) or mouse anti-human Aβ1–16 monoclonal antibody (6E10; 4C;50 ng/ml) was performed. Full length human Aβ and human Aβ fragments (singly and in combination as indicated on graph) were used to coat the plate and identify the domain that interacted with the IgG fractions following pH 7 preincubation (full length Aβ only) or incubation at pH 2.5 for 20 minutes at RT. After centrifugation and neutralization the samples were then incubated on ELISA plates coated as indicated. Values are mean ± SEM. ** P < 0.001 compared to pH 2.5 plate coated with Aβ1–42 peptide.

An epitope mapping study was performed to test if the Aβ domain specificity of the artifactual ELISA signals induced by pH 2.5 exposure differs from the domain specificity of authentic anti-Aβ antibody. ELISA plates were coated with full length sequence human Aβ1–42, N terminal human Aβ1–16, C terminal human Aβ29–40, and mixture of short human Aβ peptides as indicated on the x-axis of Fig 4. Sera from unvaccinated nonTg mice had low anti-Aβ signals with a pH 7 incubation, but an increased signal when incubated at pH 2.5. This elevation was largely specific for the full length peptide, and the signals found after pH 2.5 for other Aβ peptides or their combinations were no different than the signals seen with PBS (fig. 4A; there is also a small artifactual ELISA signal found against the plate with low pH incubation). When examining sera from mice vaccinated against Aβ (thus having authentic anti-Aβ antibodies) the low pH incubation actually reduced the apparent anti-Aβ titer due to inactivation (process B; fig 3B). Moreover, only ELISA plates containing Aβ1–16 bound the antibodies, consistent with work from our group and others that the predominant epitope for anti-Aβ vaccines is in the N terminal domain [11]. The epitope specificity of the sera from vaccinated mice was similar to that obtained with a monoclonal anti-Aβ antibody (6E10) directed against the N terminal domain (Fig 4c), again only associating when Aβ1–16 or 1–42 was bound to the plate. Thus, the artifactual binding caused by low pH incubation of normal mouse serum does not bind shorter Aβ peptides, unlike authentic anti-Aβ antibodies.

### pH 2.5 incubation induced artifactual anti-Aβ ELISA signal has a lower affinity for Aβ than authentic anti-Aβ antibodies in competition assays

**Figure 5:**
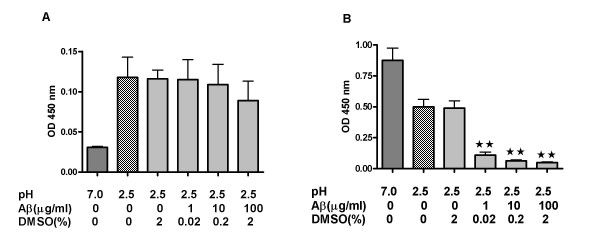
Incubation at pH 2.5 induced artifactual ELISA signal has a lower affinity for Aβ than authentic anti-Aβ antibodies in competition assays. Sera from unvaccinated mice (panel A) or mice vaccinated with Aβ 1–42 peptide (panel B), were diluted 1:1000 and preincubated at either pH 7 or pH 2.5. After centrifugation, neutralization and reconstitution of sera volume, the sera were then incubated with human Aβ1–42 peptide, at increasing concentrations at 37°C for 60 minutes. Because the Aβ was initially dissolved in DMSO, some samples were also incubated with 2% DMSO as control. The ELISA assay was then performed as described in methods with no further separation of Aβ and antibody fractions. ** P < 0.001 compared to pH 2.5 without Aβ preincubation.

To estimate the affinity of Aβ for the binding found after pH 2.5 dissociation, a competitive inhibition ELISA study was performed. Serum samples were incubated with increasing concentrations of the Aβ peptide before addition to the ELISA plate. When normal sera from nonTg unvaccinated mice were tested, the increased signal caused by incubation at pH 2.5 was unaffected by competition until Aβ concentrations were at 100 μg/ml, and even then the abrogation of signal was incomplete (fig 5A). In sera from vaccinated nonTg mice, incubation at pH 2.5 reduced the anti-Aβ ELISA signal (due to the inactivation process B), and this remaining signal was effectively competed at Aβ1–42 concentrations of 1 μg/ml and higher (fig 5B). These data indicated that the authentic anti-Aβ antibodies have at least 100 fold greater affinity for Aβ than the artifactual signal found after incubation at pH 2.5.

### Incubation at pH 3.5 avoids both the artifactual ELISA signal and the inactivation of authentic antibody signals caused by incubation at pH 2.5

**Figure 6:**
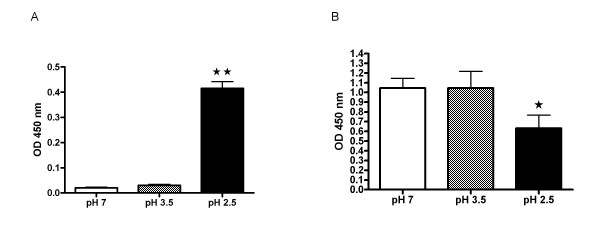
Incubation of sera at pH 3.5 does not induce artifactual ELISA signals nor inactivate authentic anti-Aβ antibody binding in ELISA assay. Sera collected from unvaccinated mice (6A) and mice vaccinated with Aβ 1–42 peptide (6B) were incubated with dissociation buffer at pH 7.0 (open bars), 3.5 (grid bars) or 2.5 (solid bars) for 20 minutes, separated by centrifugation through 10,000 MW filters neutralized and reconstituted to original volume. They were then incubated in a standard ELISA assay using full length Aβ to coat the plate. * P < 0.05 compared to pH 7.0; ** P < 0.001 compared to pH 7.0.

One possibility is that both the artifactual signal caused by low pH incubation (process A) and the inactivation of authentic anti-Aβ signals (process B) were due to excessively low pH during the incubation. Thus, we compared pH 2.5 with a slightly less aggressive incubation at pH 3.5. In normal sera collected from nonTg unvaccinated mice we found that pH 2.5 increased the apparent anti-Aβ ELISA signal, but incubation at pH 3.5 did not (fig 6A). Conversely, when sera from vaccinated nonTg mice were evaluated, incubation at pH 2.5 caused inactivation of the anti-sera raised against human Aβ 1–42 peptide (figure 5B), however, incubation at pH at 3.5 did not result in loss of anti-Aβ signal (fig 6B). Thus, neither process A not process B is apparent after incubation at pH 3.5

### Incubation at pH 3.5 rather than 2.5 can dissociate antigen antibody complexes without causing induction of artifactual signals or denaturing authentic anti-Aβ antibodies

**Figure 7:**
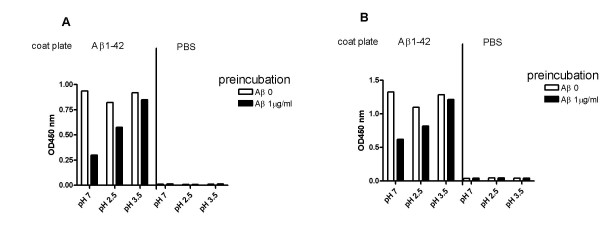
Dissociation of Aβ-IgG immune complexes occurs at both pH 2.5 and pH 3.5. Mouse anti-human Aβ1–16 monoclonal antibody (6E10) at 10 μg/ml (7A) and serum from mice vaccinated with Aβ diluted 1:100 were preincubated with Aβ1–42 peptide at 1 μg/ml to form antigen-antibody complexes (solid bars) or preincubated with PBS as a control (open bars) at 37°C for 60 minutes. Aliquots from each sample were then incubated with a 10-fold larger volume (1:1000 final serum dilution in panel B) of dissociation buffer at pH 7, pH 2.5 and pH 3.5, respectively, for 20 minutes, separated by centrifugation through 10,000 MW filters, neutralized and reconstituted to original volume for standard ELISA assay with full length human Aβ coating the plate.

To determine whether low pH does effectively unmask the anti-Aβ antibodies from Aβ (process C), we first incubated either a monoclonal antibody (fig 7A) or sera from vaccinated nonTg mice (fig 7B) with 0 or 1 μg/ml Aβ to form antigen-antibody complexes. We then subjected the samples to incubation at either a) pH 7.0, b) pH 2.5 or c) pH 3.5 and performed ELISA assays coating the plate with human Aβ1–42. Figure 7. shows that the ELISA signals of both authentic antibodies were dramatically reduced when antigen-antibody complexes were formed by mixing with 1 μg/ml Aβ and incubated at pH 7.0 (left pair of bars in 7A and 7B). Incubation at pH 2.5 (center pair of bars) had two effects; a reduction in signal intensity in the absence of antibody-antigen formation (open bars), and an increase in signal intensity when antigen antibody complexes were formed (solid bars) compared to pH 7 incubation. Importantly changing the low pH incubation to pH 3.5 avoids both issues. There is no apparent inactivation of antibody at this pH (compare open bars at pH 7 and pH 3.5) and a complete restoration of the ELISA signal when antibody-antigen complexes were formed (solid bar equals open bar at pH 3.5. In other work, we have not observed any artifactual increase in ELISA signal when sera from unvaccinated mice are incubated at pH 3.5 (data not shown).

## Discussion and conclusion

Previously, we reported that using a low pH dissociation procedure in vaccinated APP transgenic mice, anti-Aβ antibody titers increased considerably, consistent with an apparent unmasking of antibody binding sites occluded by bound antigen [5]. Unexpectedly, we subsequently found that this low pH incubation of normal sera collected from nonTg, unvaccinated mice also showed an similar increase in ELISA signals when human Aβ1–42 was used to coat the ELISA plate. Moreover, this increased signal was not observed to the same extent with other peptides including bovine serum albumin, alpha-synuclein protein, truncated Aβ11–20 and phosphate buffer saline (figure 1B). Our initial interpretation was that the untreated mouse may have anti-Aβ antibodies which were normally occluded in serum by endogenous murine Aβ. While this seemed surprising given that there was no reason to expect untreated mice to have either anti-Aβ antibodies, nor sufficient concentrations of circulating Aβ to occlude them, it was consistent with the observations we had up to that time. We also confirmed that the protein in serum that was responsible for the increased ELISA signal after low pH incubation was IgG by using more selective antibodies as reporters, and demonstrating that the increased Aβ binding was retained when IgG was purified from serum using protein A/G columns (Fig 2). Moreover cross reaction between mouse and human Aβ seemed feasible as Takeshi Kawarabayashi et al [12]showed that BNT-77, a monoclonal antibody against human Aβ11–28 could bind mouse Aβ in nonTg animals.

The interpretation that the increased ELISA signal caused by low pH incubation was due to endogenous murine anti-Aβ antibodies was challenged by differences in the behavior of this signal from untreated mice compared to that found after vaccinating nonTg mice against the Aβ peptide to generate authentic anti-Aβ antibodies. First, we observed that unlike the circumstance in transgenic mice, sera from nonTg mice vaccinated against Aβ actually lost apparent ELISA signal against Aβ when incubated at pH 2.5 (fig 3B). Epitope mapping studies found that the authentic anti-Aβ antibodies produced by vaccination bound Aβ1–16 in largely the same manner as full length Aβ, whereas, the acid-induced ELISA signal only could be detected when full length Aβ was bound to the ELISA plate (fig 4). The behavior of a monoclonal murine anti-Aβ antibody (6E10) was quite similar to the immune serum, and not the normal serum. A final inconsistency was the affinity for the Aβ peptide. Aβ at 1 μg/ml, the lowest concentration tested, largely eliminated the ELISA signal obtained with sera from vaccinated mice. However, even doses 100 fold greater, had little ability to block the increased ELISA signal found with untreated sera when incubated at pH 2.5.

Thus, whatever is responsible for the increased anti-Aβ ELISA signal found in untreated nonTg mouse serum, it has very low affinity for Aβ, and is somehow dependent upon the presence of the entire peptide to exhibit binding behavior. Given the low affinity of Aβ binding in pH 2.5 treated normal mouse serum, only serum Aβ concentrations that exceeded 100 μg/ml (25 μM concentration) would be able to mask the antibody. Given that the circulating Aβ concentration in APP overexpressing transgenic mice is 0.5 to 5 nM, it would seem this concentration is unlikely ever to occur in nonTg mice. A final argument against this hypothesis is that the nonTg mice vaccinated against Aβ do not appear to have antibody masking. Not only does the low pH fail to reveal occluded binding sites in the ELISA, there is an apparent time dependent inactivation of these authentic anti-Aβ antibodies caused by the pH 2.5 incubation. Thus we conclude that pH 2.5 incubation causes a low affinity interaction with full length Aβ that is artifactual, and unlikely to represent unmasking of authentic anti Aβ antibodies. We have referred to this as process A in the results section.

One possibility is the IgG responsible for the apparent anti-Aβ ELISA signal enhanced by low pH are natural polyreactive autoantibodies [13,14] which recognize several structurally unrelated epitopes, or relatively nonselective antibodies that recognize Aβ and a large number of similar peptide sequences with low affinity. These may be occluded by antigens other than Aβ, and become available for binding in the ELISA assays due to unmasking. However, our most favored interpretation is that the low pH partially denatures the antibodies, revealing hydrophobic residues typically shielded from interaction by their internalized location. These residues may then interact with other hydrophobic domains, such as found on the full length Aβ peptide, with low affinity but high capacity, since many IgG molecules may be susceptible to this denaturation. Such denaturation may similarly account for the loss of authentic anti-Aβ activity caused by low pH treatment of sera from vaccinated mice, the inactivation response we referred to as process B in the results.

Fortunately, the use of a less stringent method of dissociating antigen-antibody complexes can unmask occluded antibodies without a) artifactually increasing anti-Aβ ELISA signal or b) inactivating authentic anti-Aβ antibodies. Incubating at pH 3.5 instead of pH 2.5 largely eliminates the problems reported here in the acid dissociation procedure (fig 6). After spiking a serum sample with Aβ and preincubating to form antigen-antibody complexes, we observed significant loss of ELISA signal when sera were subsequently treated in a normal manner at neutral pH. However, incubating spiked sera at pH 3.5 restored the ELISA titer values to those found in the absence of added exogenous Aβ peptide (fig 7).

In our prior work (5), we now believe these three phenomena were occurring simultaneously to produce the data we observed. All of this prior work used APP transgenic mice, which would be expected to have high concentrations of circulating Aβ to bind to anti-Aβ antibodies (most of the work reported here used nonTg mice). We suspect that in addition to unmasking (process C) there were counteracting influences of authentic antibody inactivation (process B) and artifactual increases in ELISA signals (process A). in the initial work, although all three processes were active, the unmasking dominated when evaluating vaccinated APP transgenic mice that had high levels of both authentic antibodies and high levels of circulating Aβ. Only when we examined normal serum from unvaccinated nonTg mice did we reveal process A (an artifact) and only when we examined serum from vaccinated transgenic mice did we observe inactivation (process B). While we are confident that antibody masking was occurring in our earlier work and the data are accurate, our own subsequent work and reports from our colleagues indicated that pH 2.5 incubations were doing more than simply dissociating antigen-antibody complexes. Therefore we feel it important to report his improvement to our earlier technique so that others do not suffer through the same trials we have in understanding the complexities involved.

In conclusion, dissociation of antigen-antibody complexes is an important step in evaluating the anti-Aβ antibody titers in serum from APP transgenic mice, and most likely in human sera as well. This is most significant when the anti-Aβ antibody titers are low, as high titers result in excess antibody relative to Aβ peptide. Incubations at pH 2.5 cause both spurious increases in ELISA signals that are not due to high affinity anti-Aβ antibodies, and partially inactivate the ELISA signals resulting from authentic high affinity anti-Aβ antibodies probably due to denaturation. While both artifactual signals and antibody inactivation are absent at pH 3.5, antigen-antibody unmasking still occurs. This improved assay should be valuable in accurately assessing anti-Aβ antibody titers in transgenic mice and in humans treated with immunotherapy.

## Methods

### Vaccination protocols

The Tg2576 APP transgenic mice [15] and nonTg littermates were produced as described previously and have been bred continuously for over 10 years in our colony [16]. NonTg mice from this breeding were vaccinated with human Aβ1–42 peptide (American peptide) as described previously [2]. Briefly, Aβ Peptide was suspended in pyrogen-free Type I water at 2.2 mg/ml then mixed with 10× PBS to yield 1× PBS solution and incubated overnight at 37°C. The following day, two volumes of 1× PBS was added to dilute the Aβ peptide further, and then the peptide suspension was emulsified with an equal volume of Freund's complete adjuvant (Sigma). The vaccine preparation (100 μg Aβ/300 μl volume) was injected into each mouse subcutaneously. For the second immunization, the same materials were prepared freshly in incomplete Freund's adjuvant (Sigma) at 14 days after first injection. The third and fourth boosts were made incomplete Freund's at monthly intervals after the second immunization. Mice were vaccinated beginning at 9 months of age and sacrificed at 12 months of age, 14 days after the fourth inoculation.

### Dissociation of anti-Aβ antibody from Aβ in serum

Sera were diluted 1:100 with dissociation buffer (PBS buffer with 1.5% BSA and 0.2 M glycine-acetate), pH 2.5 and/or pH 3.5 to a 500 ul final volume and incubated for 20 min. at room temperature (RT). The sera were then pipetted into the sample reservoir of Microcon centrifugal filter device, YM-10 (10,000 MW cut-off; Millipore) and centrifuged at 8,000× g for 20 min. at RT. The sample reservoir was then separated from the flow through, placed inverted into a second tube and centrifuged at 1000 × g for 3 min. at RT. The collected solution containing the antibody dissociated from the Aβ peptide was adjusted to pH 7.0 with 1 M Tris buffer, pH 9.0. The retentate volume was reconstituted to the initial volume (500 μl) with ELISA dilution buffer (PBS with 1.5% BSA and 0.1% Tween-20). The collected sera were then added to an ELISA plate at several dilutions to determine the antibody titer. As control, the same serum was treated with identical process except using dissociation buffer, pH 7.0 instead of dissociation buffer, pH 2.5.

### Purification of IgG from mouse sera

The sera were pooled from four nonTg unvaccinated mice, 500 μl was added to 2 ml dissociated buffer at pH 2.5 or pH 7.0 respectively, incubated 20 minutes at room temperature then neutralized with 1 M Tri/HCL pH9.0 (pH 2.5 samples). The samples were mixed with an equal volume binding buffer (provided by ImmunoPure (A/G) IgG purification Kit, PIERCE 44902, Rockford IL USA) and then IgG were collected according to the instructions. Briefly, the diluted samples were applied to the Protein A/G Columns. After washing the Protein A/G columns with binding buffer, the IgG captured by the Protein A/G Columns were eluted by elution buffer followed by collection of successive 1 ml fractions. The eluted fractions were immediately adjusted to physiologic pH by adding 100 μl of 1 M Tris pH 9.0 to 1 ml of eluate and then the procedure for buffer exchange (desalting) was applied with 5 ml D-salt Dextran Desalting Columns. The purified IgG were measured by BCA Protein Assay Kit (PIERCE 23227).

### Measurements of antibody titers by ELISA

Ninety-six-well Immulon 4HBX plates (Dynex) were coated with 50 μl per well of human Aβ1–42 peptide 5 μg/ml in PBS buffer, pH 7.0 and incubated overnight at 4°C. The plates were washed five times with 0.45% NaCl+0.05% Tween-20 (washing buffer, WB) and blocked at 37°C for 1 hour with blocking buffer (1.5% BSA and 0.05% Tween-20 in PBS). After five washes with WB, the sera were added to the plates in duplicate and incubated for 1 hour at 37°C. The plates were washed 10 times and anti-mouse IgG conjugated with horseradish peroxidase (HRP) (Sigma Chemical Co. St. Louis, MO) diluted 1:5000 was added to the plates and incubated for 1 hour at 37°C. The plates were then washed ten times and developed with (3', 3', 5', 5'-Tetramethylbenzidine) TMB (Sigma). The reaction was stopped with 2 M sulphuric acid. The plates were analyzed spectrophotometrically at 450 nm.

### Statistical analysis

Data were analyzed by one-way analyses of variance (ANOVA), followed by Fisher's least significance difference means comparisons using StatView (SAS institute).

## Abbreviations

AD: Alzheimer's Disease

APP: Amyloid Precursor Protein

BSA; bovine serum albumin

ELISA: Enzyme linked immunosorbent assay

nonTg; nontransgenic

PBS; phosphate buffered saline

## Authors' contributions

Q-Y L prepared the vaccines and injected the mice, performed the ELISA assays and collected and analyzed the results as well as drafted the first version of the manuscript. MG generated the mice for the study, prepared the vaccines, collected the samples used for the ELISA procedures and assisted in editing the manuscript. KU and CC directed the ELISA procedures and assisted in data analysis. DM conceived the design of the study, guided data interpretation and played the major role in revision of the manuscript. All authors read and approved the final manuscript.
